# Symptom burden, quality of life, and diagnostic journey of people with postural orthostatic tachycardia syndrome, Australia, 2021–24: a descriptive patient registry data study

**DOI:** 10.5694/mja2.52710

**Published:** 2025-06-18

**Authors:** Marie‐Claire Seeley, Gemma Wilson, Eric Ong, Amy Langdon, Jonathan Chieng, Danielle Bailey, Kristina Comacchio, Amanda Page, Dennis H Lau, Celine Gallagher

**Affiliations:** ^1^ Australian Dysautonomia and Arrhythmia Research Collaborative the University of Adelaide Adelaide SA; ^2^ South Australian Health and Medical Research Institute Adelaide SA; ^3^ The University of Adelaide Adelaide SA; ^4^ Royal Adelaide Hospital Adelaide SA

**Keywords:** Autonomic nervous system diseases, Chronic disease, Diagnosis, SARS‐CoV‐2

Postural orthostatic tachycardia syndrome (POTS) is a multisystem autonomic disorder; its aetiology is poorly understood, and it is associated with significant disability.^1^ In this article, we report our analysis of Australian POTS Patient Registry data, with the aim of exploring the symptom burden, quality of life, and diagnostic journey of people in Australia with POTS.

For our descriptive study, we included data for people aged 16 years or older with clinically diagnosed POTS who were enrolled in the Australian POTS registry during 1 May 2021 – 30 April 2024 following review at a specialist POTS clinic in Adelaide. The confirmed diagnosis was based on international criteria, including a sustained heart rate increase of at least 30 beats per minute or at least 40 beats per minute in adolescents (16–19 years of age) during a 10‐minute active standing test or head‐up tilt table test, the absence of orthostatic hypotension, and unexplained symptoms of orthostatic intolerance lasting at least three months.^2^ Prior to their first clinical review, autonomic symptom burden (Composite Autonomic Symptom Score, COMPASS‐31)^3^ and quality of life (a United Kingdom EuroQol EQ‐5D‐5L value set was used to generate a health‐related quality of life utility score, range 0 to 1, with 1 = full health; and a visual analogue score, range 0 to 100, with 100 = best imaginable health^4^) were assessed for each person via electronic link to a REDCap database.^5^ We summarise demographic and clinical characteristics as means with standard deviations (SDs). The statistical significance of between‐group differences was assessed in independent samples *t* tests (continuous variables) and χ^2^ tests (categorical variables); *P* < 0.05 was deemed statistically significant. All statistical analyses were performed in SPSS Statistics 28.0.1.0. The study was approved by the University of Adelaide Human Research and Ethics Committee (H‐2021‐052) and was conducted in accordance with the 2024 Declaration of Helsinki standards for human research. All participants provided informed consent prior to enrolment in the POTS registry for the use of their data in published research.

The registry included data for 500 people with POTS; their mean age was 31.3 years (standard deviation [SD], 11.7 years), 434 were women (86.8%), 483 were white (96.6%), and 76 were adolescents (15.2%). Diagnostic delays of more than ten years had been experienced by 133 people (25.5%); 110 were unemployed (22.0%) and 130 had social outings less frequently than once per month (26.0%) (Box). The mean diagnostic delay was longer for women (7.0 [SD, 8.6] years) than for men (3.8 [SD. 5.4] years; *P* = 0.010). The mean diagnostic delay for people diagnosed during 2019–2024 was shorter than for those diagnosed before 2019 (1.6 [SD, 1.6] years *v* 12.9 years, [SD, 9.2] years; *P* < 0.001). Infection was the most frequent trigger of POTS symptom onset (197 people, 39.4%), including 163 with POTS secondary to severe acute respiratory syndrome coronavirus 2 (SARS‐CoV‐2) infections (32.6%) (Box).

Emergency department presentations related to POTS symptoms prior to their diagnosis with POTS were recorded for 284 people (54.5%; median, 3 [interquartile range, 1–5] visits); the physical symptoms of 323 people (64.6%) had been attributed to anxiety prior to their diagnosis. People had seen a mean of 5.2 (SD, 4.6) physicians prior to diagnosis, and were currently consulting a mean of 7.5 (SD, 4.5) specialists (Box); 65 (13.0%) had consulted more than ten doctors before being diagnosed with POTS.

Box. 1 **Socio–demographic characteristics, diagnosis history, and triggers of disease for 500 people enrolled in the Australian Postural Orthostatic Tachycardia Syndrome Patient Registry, Adelaide, 1 May 2021 – 30 April 2024**

**Table:** 

Characteristic	Value
Socio‐demographic characteristics	
Age (years), mean (SD)	31.3 (11.7)
Adolescents (16–19 years)	76 (15.2%)
Sex (women)	434 (86.8%)
Ethnic background (white)	483 (96.6%)
Tertiary education	341 (65.6%)
Fulltime work	91 (18.2%)
Unemployed	110 (22.0%)
Social outings less than once per month	130 (26.0%)
Body mass index (kg/m^2^), median (IQR)	24.2 (21.1–28.7)
Diagnosis history	
Age at symptom onset in adults (years), median (IQR)	22.0 (15.0–33.0)
Age of symptom onset in adolescents (years), median, (IQR)	14.0 (12.2–16.0)
Symptom onset after 18 years of age	294 (56.4%)
Diagnostic delay (years), mean (SD)	6.7 (8.4)
Diagnostic delay longer than ten years	133 (25.5%)
Number of physicians seen prior to diagnosis, mean (SD)	5.2 (4.6)
Number of specialists currently seen, mean (SD)	7.5 (4.5)
Symptoms attributed to anxiety prior to diagnosis	323 (64.6%)
Presented to emergency department with symptoms prior to diagnosis	284 (54.5%)
Onset trigger	
Infection	197 (39.4%)
Trauma/concussion	34 (6.8%)
Vaccination	29 (5.8%)
Surgery	17 (3.4%)
Other*	44 (8.8%)
No identifiable trigger	179 (35.8%)

IQR = interquartile range, SD = standard deviation.

* Pregnancy, eating disorder, stress event, puberty, allergic reaction, menopause.

Total COMPASS‐31 scores and its secretomotor, vasomotor, and pupillomotor component scores were similar for adolescents and adults (data not shown). However, mean orthostatic intolerance was greater for adolescents than adults (29.9 [SD, 6.8] *v* 27.6 [SD. 7.7]; *P* = 0.026); mean bladder (1.3 [SD, 1.7] *v* 1.8 [SD, 2.1]; *P* = 0.012) and gastrointestinal symptom scores (8.9 [SD, 4.8] *v* 10.5 [SD, 4.4]; *P* = 0.007) were less severe for adolescents than adults. The mean EQ‐5D‐5L scores were similar for adolescents and adults (data not shown).

The overall mean health‐related quality of life utility score was 0.591 (SD, 0.240), the mean visual analogue scale score was 45.9 (SD, 20.8). “Usual activities” (347 people, 69.4%) and “pain and discomfort” (318 people, 63.6%) were the EQ‐5D‐5L subdomains in which moderate to severe problems were most frequent.

This is the first Australian study to report data from a patient registry of people with physician‐confirmed POTS. Our key findings include long delays between symptom onset and diagnosis despite frequent health care interactions, and reduced social engagement, high unemployment, reduced ability to undertake usual activities, and low quality of life for people with POTS, despite their relatively young age.

The impact on quality of life was similar for adolescents and adults, but the symptom burden was different. We found that mean orthostatic intolerance was greater in adolescents than adults, but bladder and gastrointestinal symptoms were more severe in adults. These differences suggest either a distinct presentation or progression of autonomic dysfunction in adolescents and adults, which should be further investigated. Notably, the quality of life was similar for both age groups despite these differences.

The mean diagnostic delay in Australia was longer than reported overseas,^6^ suggesting specific barriers in Australian health care. Our findings raise concerns about the broader socio‐economic impact of POTS, given the reported increase in the risk of POTS after SARS‐CoV‐2 infection.^7^, ^8^ While the mean diagnostic delay was lower during 2019–24 than prior to 2019, suggesting clinical recognition is at least improving in South Australia, our findings reflect a cohort that consisted of people who could access care at a specialist clinic, rather than equitable access across Australia to an appropriate assessment and treatment pathway. Further, most of the people in our study were white, as in most overseas reports, raising concerns about diagnostic inequities for people from culturally and linguistically diverse groups; it is likely that many people with POTS remain undiagnosed.

Research that informs clinical management by improving understanding of the aetiology of POTS and evaluates treatment options is urgently needed to reduce the disability associated with this condition. Targeted interventions, better primary health care, education, and care pathways tailored to the younger women who comprise the majority of people with POTS are essential for reducing the number of acute care presentations. Longitudinal research, with diverse patient groups and treatment‐focused approaches, is needed to improve outcomes.

## Open access

Open access publishing facilitated by the University of Adelaide, as part of the Wiley – the University of Adelaide agreement via the Council of Australian University Librarians.

## Competing interests

Marie‐Claire Seeley has received consulting fees from Argenx, paid to the Australian POTS Foundation. Dennis Lau has received lecture and consulting fees from Abbott Medical, Biotronik, Medtronic, and MicroPort CRM, paid to the University of Adelaide.

## Data sharing

The de‐identified data we analysed are not publicly available, but requests to the corresponding author for the data will be considered on a case‐by‐case basis.

Received 23 September 2024, accepted 24 March 2025
